# Design and Characterization of Dicyanovinyl Reactive Dyes for the Colorimetric Detection of Thiols and Biogenic Amines

**DOI:** 10.3390/s18030814

**Published:** 2018-03-08

**Authors:** Tinkara Mastnak, Aleksandra Lobnik, Gerhard J. Mohr, Matejka Turel

**Affiliations:** 1 Faculty of Mechanical Engineering, University of Maribor, Smetanova 17, SI-2000 Maribor, Slovenia; tinkara.mastnak@um.si (T.M.); aleksandra.lobnik@um.si (A.L.); 2 Institute for Environmental Protection and Sensors, Beloruska 7, SI-2000 Maribor, Slovenia; 3 JOANNEUM RESEARCH Forschungsgesellschaft mbH—Materials, Franz-Pichler-Straße 30, A-8160 Weiz, Austria; gerhard.mohr@joanneum.at

**Keywords:** indicator dye, chromoreactand, absorption, thiols, biogenic amines

## Abstract

The synthesis of two new azobenzene dyes, namely CR-528 and CR-555, and their spectral properties in ethanol solution are described. The recognition of sulfur-containing analytes (2-mercaptoethanol (2-ME), sodium hydrosulfide (NaHS)), and biogenic amines (spermine, spermidine, ethanolamine) bestowed significant spectral changes with color changes from pink/purple to pale yellow/orange-yellow. The nitro acceptor group in the dicyanovinyl reactive dye contributes to higher sensitivity and lower detected analyte concentrations. The absorption maxima of both the dyes are at wavelengths compatible with low-cost light sources and detectors, making them excellent candidates for optical probes that are economic, simple to use, and do not require well-trained personnel.

## 1. Introduction

Biologically derived thiols (biothiols) are important antioxidants that protect cells from oxidative damage. They are involved in signal transduction, apoptosis, and protein structure. Changes in their levels have been correlated to a variety of medical disorders, including liver damage, osteoporosis, cardiovascular diseases, stroke, diabetes mellitus, alcoholic cirrhosis, and Alzheimer’s [1]. In addition, some thiol compounds were related to plant development [2] and their tolerance to abiotic stress [3]. Many studies also reported the relevance of volatile thiols as aroma components in foodstuffs such as cheese, wine, beer and even coffee [4].

Biogenic amines are nitrogen-containing low molecular weight organic compounds that are naturally synthesized in animals, plants, and microorganisms—generally by decarboxylation of free amino acids or by amination or transamination of aldehydes and ketones [5]. Certain biogenic amines, like spermine and spermidine, are among the most abundant organic polycations that are found in the human body, involved in protein synthesis, conformational stabilization of nucleic acids, and cytoskeleton structures, and they play multiple roles in cell growth, proliferation, and survival [6,7]. Consequently, changes in levels of spermine and spermidine have been associated with aging and diseases, like cancer [8]. Furthermore, they have been found to be growth regulators in plants required for response to various stress factors [9]. The presence of spermine and spermidine is also considered a marker for food quality, since their concentration increases due to fermentation or spoilage [10].

Owing to their important roles, sensitive and selective detection of biogenic thiols and amines has received growing attention in recent years. A variety of methods have been reported for this purpose, such as gas chromatography (GC), GC coupled with mass spectrometry (GC/MS) or high performance liquid chromatography (HPLC). These methods are specific and accurate but expensive, time-consuming, and need to be performed by well-trained personnel [4,11].

Metal oxide sensors are extensively used as gas sensors. The sensing approach is based on oxide semiconductor materials, such as SnO_2_, ZnO, or TiO_2_, serving as chemiresistors, which show different conductivities upon interaction with the analyte gas. Metal oxide sensors were also successfully used for detection of amines [12,13,14] and volatile sulfur compounds [15,16]. In this context, electronic nose systems have been widely tested for quality control of products in the food and aroma industries. These instruments mimic the human olfactory perception through an array of chemical sensors (e.g., metal oxide semiconductor sensors) with partial specificity and overlapping sensitivity. When combined with a suitable signal processing unit, they are able to detect and distinguish odors, including certain amines and thiols [17].

Optical methods, such as UV-Vis or fluorescence spectroscopy, are particularly attractive as analytical tools due to their simplicity, sensitivity, low cost, ease of miniaturization, and potential for in-situ measurement. Accordingly, they have been used for determination of thiols like cysteine, homocysteine, glutathione, H_2_S [18,19,20,21,22,23,24,25,26,27], and (biogenic) amines, namely phenethylamine, tyramine, histamine, ethylamine, methylamine, agmatine, isopentylamine, propylamine, and putrescine [28,29,30,31,32,33,34,35,36], by using specific indicator dyes that exhibit different spectral characteristic when exposed to these analytes. To mention some of the indicator dyes used for the detection of thiols, Deng et al. developed a colorimetric and ratiometric fluorescent probe with diketopyrrolopyrrole (DPP) fluorophore, which (in the presence of cysteine) changed from purple to yellow, whereas the fluorescence changed from red to yellow [37]. Another naked-eye method with colorimetric and fluorometric readouts for detecting l-cysteine in solution was proposed by Fu et al. They used rhodamine B-functionalized gold nanoparticles (RB-AuNPs) coupled with the nanometal surface energy transfer (NSET) technique [38]. To detect cysteine, Khajehsharifi and Sheini used Zincon complexed with Zn^2+^ ions in an indicator displacement assay, where adding the analyte to solution of the Zincon–Zn^2+^ complex resulted in a ligand exchange between Zincon and cysteine. The reaction was accompanied by an obvious color change of the solution from blue to orange-yellow [39]. As an example of colorimetric and ratiometric fluorescent probes for glutathione (GSH) at physiologically relevant concentration, Zeng et al. used a naphthalene-2,3-diamine derivate containing piazselenole. Upon the addition of GSH, the probe displayed a ratiometric fluorescent response with an enhancement of the ratios of emission intensities at 436 and 615 nm, accompanied with the color change from orange to colorless [40]. The aldehyde function of 4-*N*,*N*-dioctylamino-4′-formyl-2′-nitroazobenzene was found to provide significant color changes with hydrogen sulphite [41], but later also to be responsive to cysteine and homocysteine [42]. For the detection of biogenic amines in solution, Malik et al. synthesized the water-soluble cationic conjugated polymer [9,9-bis(6′-methylimidazoliumbromide)hexyl)-fluorene-co-4,7-(2,1,3-benzothiadiazole)] (PFBT-MI) and combined it with a surfactant to achieve aggregates, which enabled the detection of spermine [6]. Tyrosine-protected gold nanoparticles (Tyr-Au NPs) were used by Rawat et al. as a dual probe for colorimetric and fluorescence turn-on assays of spermine and spermidine in biological samples. Upon the addition of spermine and spermidine, the aggregation of Tyr-Au NPs was induced, which resulted in a red-shift and fluorescence turn-on [29]. Moreover, Chopra et al. processed a Schiff-base receptor into organic nanoaggregates and prepared its Cu^2+^ complex. This was further employed as a sensor for detection of biogenic amines in aqueous medium, and showed selective sensing of spermine, where upon its addition to the complex, the color changed from yellow to blue [43]. A tricyanovinyl azobenzene dye has been shown to be sensitive to aliphatic amines (ethylamine, 1-propylamine, 1-butylamine, diethylamine, triethylamine), both in a solution and incorporated into polymer layers [44], whereas the tricyanoethylene derived colorimetric chemodosimeter was used to detect hydrogen sulfide [45].

Colorimetric detection methods have relatively undemanding requirements; therefore, the aim of this study was to synthesize and characterize new probes for detection of sulphur and amine-containing analytes that enable naked-eye detection and are suitable for usage with cheap light sources. Suitably large spectral changes between the off and on state of a colorimetric system allow for unassisted visual detection, sometimes even in the form of indicator papers and test strips [46]. Simple detection methods are useful for applications in which instrumentation or laboratory costs are often prohibitive, or otherwise unavailable, e.g., in underdeveloped countries. Our newly synthesized azobenzene dyes CR-528 and CR-555 show significant spectral changes from pink or purple, respectively to pale yellow or orange yellow when exposed to increasing concentrations of sulfur-containing analytes (2-mercaptoethanol (2-ME), sodium hydrosulfide (NaHS)) and biogenic amines (spermine, spermidine, ethanolamine) in ethanol solution. A comparison with other developed probes that are sensitive to similar analytes is given, showing the advantages of CR-528 and CR-555 over these probes in terms of working range and response time. Moreover, the absorption maxima of both dyes are present at wavelengths compatible with low-cost light sources and detectors. Thus, they may become probes used as sensor receptors in optical sensor devices that are economic, simple to use, and do not require well-trained personnel.

## 2. Materials and Methods

### 2.1. Chemicals and Starting Materials

All of the amines and 2-ME were of analytical grade and obtained from Sigma (Maribor, Slovenia). NaHS was of technical grade and obtained from MOLEKULA (Ljubljana, Slovenia). Absolute ethanol was obtained from Panreac (Ljubljana, Slovenia). All of the chemicals for synthesis were obtained from Aldrich (Vienna, Austria).

### 2.2. Physical Measurements

Melting points were measured with the MPA120 melting point apparatus (Stanford Scientific, Ljubljana, Slovenia). Elemental analysis was measured with 2400 Series II CHNS/O elemental analyser (Perkin-Elmer, Ljubljana, Slovenia). NMR spectra were obtained in CDCl_3_ using a Bruker Avance III 300 MHz NMR spectrometer (Ljubljana, Slovenia) with chemical shifts reported in parts per million (ppm) relative to tetramethylsilane (TMS). High resolution mass spectra (HRMS) were recorded on an Agilent 6224 Accurate-Mass TOF LC/MS system (Vienna, Austria).

### 2.3. Spectrophotometric Measurements

Absorption spectra were obtained with a Lambda 35 UV-Vis spectrometer (Perkin-Elmer, Ljubljana, Slovenia) at 20 ± 1 °C. All spectra were obtained in polystyrene cuvettes (path length 1 cm) purchased from Ratiolab (Maribor, Slovenia).

General procedure for spectrophotometric measurements consisted of preparing stock solutions of both dyes and all the analytes in absolute ethanol. The CR-528 stock solution had concentration of 7.2 × 10^−6^ M (M denotes mol/L) and the CR-555 stock solution a concentration of 7.9 × 10^−6^ M. Test solutions were prepared by placing 2.9 mL of the CR-528 or CR-555 stock solution into a cuvette and adding 0.1 mL of the analyte solution with the desired concentration. Each cuvette was sealed with a polypropylene stopper to prevent evaporation. The spectra typically were recorded after 30 min incubation time.

### 2.4. Synthesis

**Figure 1:**
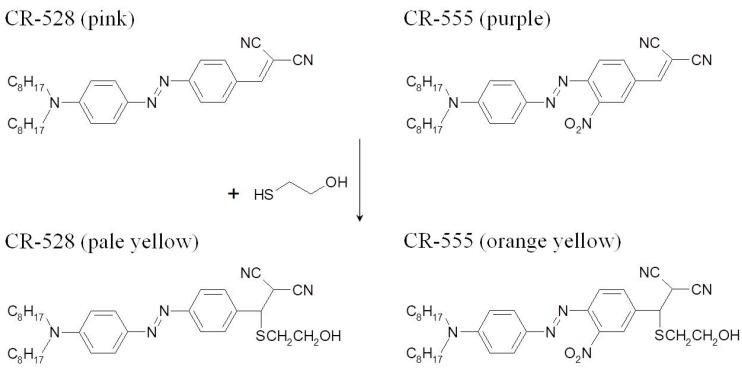
Chemical structures of the azobenzene dyes CR-528 and CR-555 before and after the reaction with 2-ME.

The chemical structures for both azo dyes are shown in Figure 1 together with the pathway [27] of interaction with thiols. Their structures were confirmed using NMR, HRMS (ESI-TOF), and elemental analysis.

#### 2.4.1. 4-*N*,*N*-dioctylamino-4′-dicyanovinylazobenzene (CR-528)

1 g (5.9 mmol) of 4-dicyanovinylaniline [47,48] was dissolved in 12 mL of acetic acid, following the addition of 0.95 mL (17.8 mmol) of concentrated sulfuric acid. This solution was cooled to 5 °C and 0.38 g (5.5 mmol) of sodium nitrite that was dissolved in 1 mL of water was added slowly under vigorous stirring. The solution was heated to room temperature and stirred for another fifteen minutes. Then, the diazonium salt solution was added to 1.9 g (6.0 mmol) of *N*,*N*-dioctylaniline [49] in 50 mL of acetic acid at 10 °C. After 30 min, 3 g (22 mmol) of sodium acetate trihydrate dissolved in 4 mL of water was added and the mixture was stirred at 10 °C overnight. The resulting mixture containing the lipophilic dicyanovinylazo dye CR-528 was diluted with 150 mL of water, extracted into 100 mL of dichloromethane, washed three times with 50 mL of water, and dried over magnesium sulfate. The azo dye was then purified using 90 g of silica gel on a 5 cm diameter column, with dichloromethane/hexane = (2:1) as the eluent. 1H-NMR (250 MHz, CDCl_3_) of 4-*N*,*N*-dioctylamino-4′-dicyanovinylazobenzene (CR-528), δ (ppm): 8.00 (d, 2H), 7.89 (m, 4H), 7.74 (s, 1H), 6.70 (d, 2H), 3.39 (t, 4H), 1.65 (m, 4H), 1.34 (m, 20H), 0.90 (t, 6H). Calculated for C_32_H_43_N_5_ (497.41): C, 77.27; H, 8.71; N, 14.08, found C, 77.40; H, 8.69; N, 14.06. HRMS (ESI-TOF) calcd. for C_32_H_43_N_5_ [M+H^+^] 498.3593, found 497.3521, M.p. 80–82 °C.

#### 2.4.2. 4-*N*,*N*-dioctylamino-2′-nitro-4′-dicyanovinylazobenzene (CR-555)

4-Dicyanovinyl-2-nitroaniline (0.7 g, 3.3 mmol) [50,51] was dissolved in 8 mL of acetic acid. Then, 0.7 mL (13.1 mmol) of concentrated sulfuric acid was added, following the addition of 0.22 g (3.1 mmol) of sodium nitrite in 0.5 mL water. Again, coupling was performed to *N*,*N*-dioctylaniline (1.2 g, 3.8 mmol) in acetic acid (20 mL). After the addition of 1.5 g (11 mmol) of sodium acetate trihydrate in 2 mL of water, and the mixture was stirred at 10 °C overnight. The resulting lipophilic dicyanovinylnitroazo dye CR-555 was extracted from the mixture as described above, and purified by column chromatography using dichloromethane/hexane = (2:1) as the eluent. 1H-NMR (250 MHz, CDCl_3_) of 4-*N*,*N*-dioctylamino-4′-dicyanovinyl-2′-nitroazoben-zene (CR-555), δ (ppm): 8.20 (m, 2H), 7.92 (m, 3H), 7.73 (s, 1H), 6.71 (d, 2H), 3.43 (t, 4H), 1.67 (m, 4H), 1.33 (m, 20H), 0.92 (t, 6H). Calculated for C_32_H_42_N_6_O_2_ (542.73): C, 70.82; H, 7.80; N, 15.49, found C, 70.03; H, 6.99; N, 15.87. HRMS (ESI-TOF) calcd. for C_32_H_42_N_6_O_2_ [M+H^+^] 543.3447, found 542.3375, M.p. 91–93 °C.

## 3. Results and Discussion

### 3.1. Spectral Properties of CR-528 and CR-555 in Ethanol

**Figure 2:**
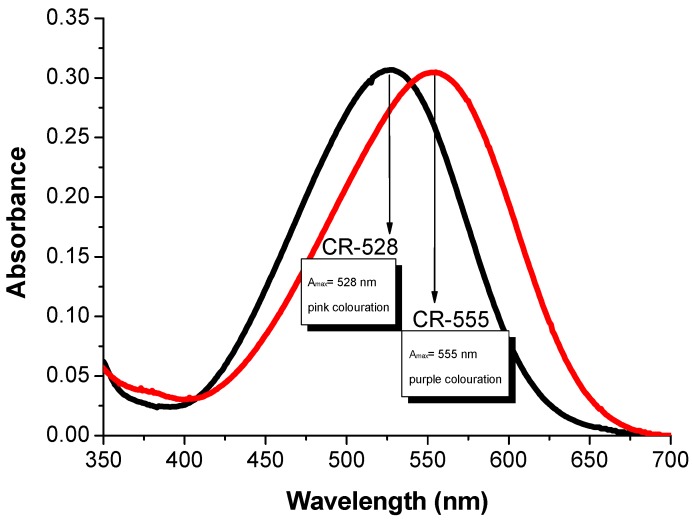
Absorption spectra of CR-528 (2.4 × 10^−7^ M) and CR-555 (2.6 × 10^−7^ M) in ethanol.

The absorption spectrum of CR-528 exhibits its maximum intensity at 528 nm with a molar extinction coefficient (ε) of 42800 M^−1^cm^−1^, resulting in a pink coloration (Figure 2). This strong absorption is due to the pronounced donor and acceptor strengths of the substituents that were attached to the azobenzene structure (Figure 1). The dicyanovinyl group exhibits a Hammett substituent constant σp of 0.84 which is comparable to the nitro group (σp of 0.78), while the diethylamino function has a σp of −0.72 [52]. In contrast, CR-555 in ethanol solution is purple and shows an absorption maximum at 555 nm (ε = 39200 M^−1^cm^−1^), due to the combined effect of the dicyanovinyl and the nitro acceptor groups. Both of the dyes were dissolved in other solvents too (Figure S1), where solvatochromic effects were more pronounced in the case of CR-528. Nevertheless, ethanol was chosen for our further experiments because analytes of interest are soluble in this particular solvent.

### 3.2. Optical Response of CR-528 and CR-555 towards 2-ME

**Figure 3:**
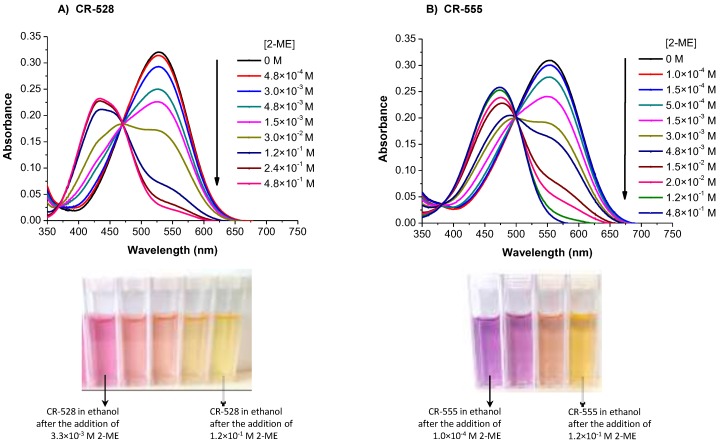
Absorption spectra of CR-528 (2.4 × 10^−7^ M; (**A**)) and CR-555 (2.6 × 10^−7^ M; (**B**)) in the presence of various concentrations of 2-ME (from 0 to 4.8 × 10^−1^ M) in ethanol solution.

To explore the potential sensing properties of both azo dyes, we first conducted experiments with 2-ME. When an ethanol solution of CR-528 was reacted with increasing concentrations of 2-ME, then the absorbance maximum at 528 nm decreased and a new maximum was formed at 434 nm, as shown in Figure 3a, which caused a significant color change from pink to pale yellow. The exposure of a CR-555 solution to increasing concentrations of 2-ME was accompanied by a decrease in absorption at 555 nm and the formation of a shifted maximum at 474 nm, as shown in Figure 3b, corresponding to a color change from purple to orange yellow. Figure 1 shows chemical structures of both dyes before and after reaction to 2-ME. The nucleophilic thiol performs an irreversible addition reaction to the ethene group of the dicyanovinyl receptor, as suggested by Kwon et al. [27]. In both cases, the spectra (Figure 3) distinctly show isosbestic points which indicate a single chemical reaction to take place. Since both color changes were clearly visible, both azo dyes can serve as a ‘‘naked-eye’’ indicator for 2-ME.

**Figure 4:**
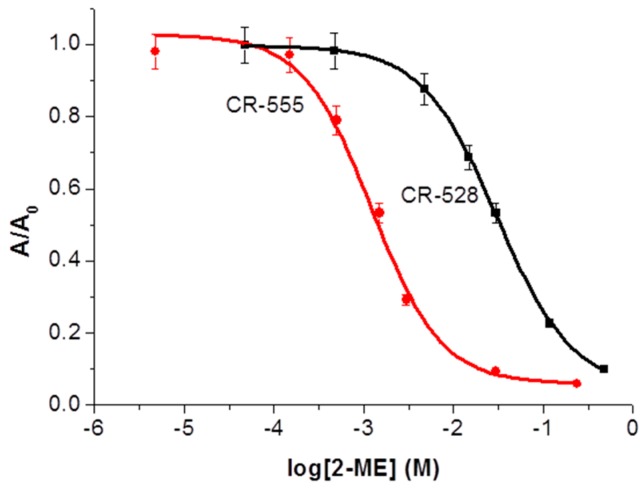
Spectrophotometric titrations of CR-528 and CR-555 in ethanol as a function of mean normalized absorbance maximum (taken at 528 nm for CR-528 nm and at 555 nm for CR-555). Each data point represents an average value of three measurements. Error bars show standard error.

Calibration plots in Figure 4 show that the affinity of CR-555 for determining 2-ME is higher when compared to CR-528 dye, as CR-555 can determine about 20-fold lower concentrations of 2-ME. The concentration range in the case of CR-555 probe spans from 1.5 × 10^−4^ to 1 × 10^−2^ M for 2-ME, whereas in the case of CR-528 probe, the concentration range is between 3 × 10^−3^ to 3 × 10^−1^ M for the same analyte. The higher affinity of CR-555 to 2-ME is attributed to the dye’s higher reactivity due to the presence of a nitro group.

### 3.3. Time-Dependent Absorbance Changes of CR-528/CR-555 upon Exposure to 2-ME in Ethanol

**Figure 5:**
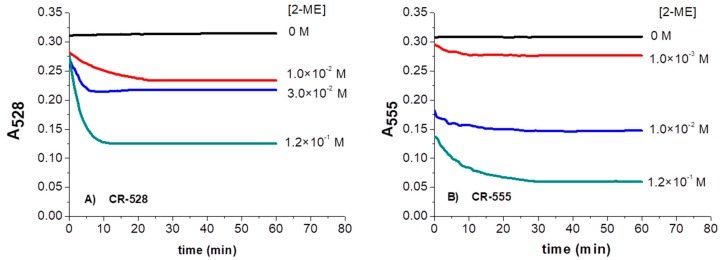
Time-courses of CR-528 (2.4 × 10^−7^ M; (**A**)) and CR-555 (2.6 × 10^−7^ M; (**B**)) in the presence of various concentrations of 2-ME (from 0 to 1.2 × 10^−1^ M) in ethanol solution, recorded at A_528_ and A_555_, respectively.

To understand how the optical properties of CR-528 and CR-555 changed with time upon interaction with 2-ME, the intensity at maximum absorbance in the presence of various concentrations of 2-ME over 60 min was recorded (Figure 5). The results for CR-528 show that the absorption intensity readily decreased at 528 nm and the reaction was completed after around 30 min.

A similar behavior was observed for CR-555. The chemical reaction with 2-ME was also completed within 30 min. In the case of chromoreactand CR-555, larger absorbance changes in the presence of similar concentrations of 2-ME were observed when compared to CR-528, due to the higher chemical reactivity of the dicyanovinyl group in CR-555.

### 3.4. Optical Response of CR-528 and CR-555 towards Sulfur- and Amine-Analytes

Ethanol soluble sulfur and biogenic amine species were chosen to further explore the sensitivity of the chromoreactands CR-528 and CR-555. NaSH and the biogenic amines tyramine, cadaverine, putrescine, histamine, ethanolamine, spermine, and spermidine were used to evaluate the reactivity of CR-528 and CR-555. Amine solutions of 1.0 × 10^−2^ M were added to CR-528/CR-555 stock solutions, and the same experiment was carried out with NaSH for comparison with 2-ME. Significant color changes were noted in all cases, except in the case of putrescine and cadaverine. Among the tested biogenic amines, spermine and spermidine were finally chosen for further experimental work due to their importance as markers of different types of cancer [53]. Ethanolamine was considered too, due to its structural similarity to 2-ME. The spectral responses of CR-528 and CR-555 to tyramine, histamine, cadaverine, and putrescine are shown in Figures S2 and S3.

**Figure 6:**
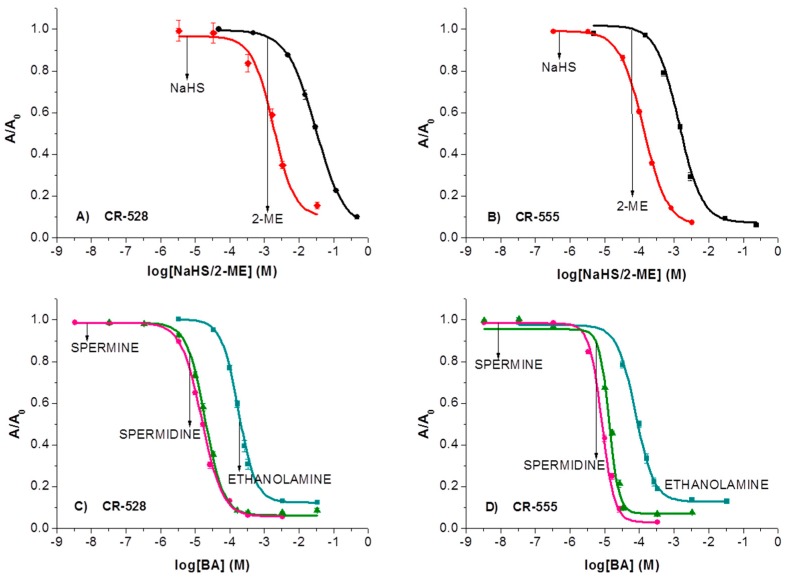
Spectrophotometric titrations (calibration curves) for sulfur-based analytes (NaHS, 2-ME; (**A**,**B**)) and for amine-based (BA) analytes (spermine, spermidine, ethanolamine; (**C**,**D**)); *n* = 3.

When ethanol solutions of either CR-528 or CR-555 were reacted with increasing concentrations of NaHS, ethanolamine, spermine, and spermidine, we observed similar color changes and response as in the case of 2-ME. Figure 6 shows spectrophotometric titrations (calibration curves) for sulfur compounds (Figure 6A,B) and the selected-biogenic amines (Figure 6C,D). The curves are presented in semilogarithmic scale with absorbance values as A/A_0_, where A is the absorbance in the presence of the analyte and A_0_ is the absorbance in the absence of the analyte. Each point of the calibration curve is a result of three independent measurements after 30 min incubation time.

Additionally, Table 1 gives all λ_max_ values and X_0_ values statistically obtained from Boltzmann sigmoidal models for the presented analytes. The inflection point values of all plots of absorbance versus concentration (X_0_) were used to compare the reactivity of the analytes. Figure 6 and Table 1 show that 4–10 fold higher sensitivity can be attributed to the chromoreactand CR-555 when compared to CR-528. Furthermore, the overall sensitivity of both chromoreactands is approximately one order of magnitude higher for amines than for sulfur compounds. This stems from the better nucleophilic character of amines compared to sulfur compounds, so stronger nucleophilicity of the analyte results in increased sensitivity and lower detection limit. The reaction between the chromoreactands and the analytes also differs in the absorption maxima, caused by the different chemical structures of the indicator dyes.

As already found by Kwon et al. [27] nucleophiles, such as amines and thiols, interact with dicyanovinyl groups and perform an irreversible addition reaction. This affects the acceptor strength of the dicyanovinyl group, causing the colour of the chromophore to change (see Figure 1). Similar irreversible chemical reactions, albeit of tricyanovinyl groups, have already been shown to provide indicator dyes for lipophilic amines and sulfur containing amino acids [44,45].

### 3.5. Comparison with Developed Probes for the Optical Determination of Relevant Sulfur-Containing Analytes and Amines

In Table 2 the comparison data of developed probes for the optical determination of the relevant sulfur-containing analytes and biogenic amines are listed, along with chromoreactands CR-528 and CR-555 properties optimized within this work. However, there is little related literature and the experiments for probes by various labs were conducted in different solvent systems using different indicators, so the developed probes are not directly comparable.

One can see that the use of absorbance and fluorescence-based techniques are equally distributed. Coumarin–malonitrile conjugate [27] suffers from a rather long response time of 330 min. Cu(II) complex of Schiff-base receptor organic nanoaggregates [43] is the only probe that showed lower response time than CR-528 and CR-555, but it enables the detection of a single analyte. Working ranges are mainly up to two decades. Wider ranges than 2 decades have the systems based on PFBT-MI and surfactant [6] and Tyrosine-protected gold nanoparticles [29]. CR-528 and CR-555 offer wider working range for the detection of NaSH than the fluorescent probe HF-PBA [26] and 4-chloro-7-nitrobenzofurazan [25] do for H_2_S. Apart from Tyrosine-protected gold nanoparticles [29], the chromoreactands provide the lowest working range for the detection of spermine and spermidine. However, the cost of synthesizing CR-528 and CR-555 is only a fraction of what it costs to synthesize gold nanoparticles.

When compared to current probes for determination of sulfur-containing analytes and biological amines, the newly synthesized dyes, namely CR-528 and CR-555, can be synthesized easily without any complicated processes. Moreover, as the probes display colorimetric response with distinct color change, they are more suitable for practical determination due to compatibility with cheap light sources. In addition, no other probe enables the visual detection of both species (biological thiols and bioamines). The results of the preliminary research with newly synthesized chromoreactands CR-528 and CR-555 can provide a new platform for visual quantification of biological thiols and bioamines, which may be a useful tool in several fields of application. Although additional methods are beginning to emerge, new detection strategies are greatly needed.

## 4. Conclusions

Two new azobenzene dyes, CR-528 and CR-555, have been synthesized and their spectral properties in ethanol solution have been investigated. The recognition of sulfur/thiol-containing analytes and biogenic amines caused significant spectral changes, corresponding to color changes from pink/purple to pale yellow/orange-yellow. These concentration-dependent color changes can readily be interpreted by the eye, but are also compatible with low-cost light sources and detectors. Due to the presence of the NO_2_ group on CR-555, the reactivity of this chromoreactand is higher when compared to CR-528 without nitro group. Consequently, CR-555 enables the determination of lower concentrations of sulfur- and amine-based analytes and shows that proper synthesis enables tailoring the sensitive range to certain extent.

Further investigations will focus on the development of optical sensor materials based on the incorporation of CR-528 and CR-555 into polymer layers. Then, we plan to detect biological thiols in solution at physiological pH (7.4). At these conditions, the amines are found in their protonated form, and are therefore unreactive; consequently, selectivity of such probes will be governed by the pH of the sample solution. Additional interfering thiol and amine species will be easily tested as in this study only ethanol soluble analytes were able to be checked.

## Figures and Tables

**Table 1:** Analyte structures and their reactivity with CR-528 and CR-555.

Analyte	Analyte Structure	CR-528		CR-555	
		X_0_^a^ (M)	λ_max_^b^ (nm)	X_0_^a^ (M)	λ_max_^b^ (nm)
2-ME	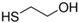	3.00 × 10^−2^	434	1.35 × 10^−3^	474
Sodium hydrosulfide	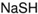	1.66 × 10^−3^	390	1.29 × 10^−4^	464
Ethanolamine	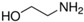	1.73 × 10^−4^	460	1.37 × 10^−5^	490
Spermine	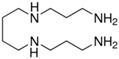	1.46 × 10^−5^	437	8.37 × 10^−5^	475
Spermidine	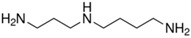	2.03 × 10^−5^	443	8.77 × 10^−6^	469

^a^ inflection point; ^b^ A_max_ of synthesized dyes in the presence of the respective analyte.

**Table 2:** Comparison data of developed probes for the detection of relevant sulfur-containing analytes and biogenic amines.

Indicator	Analyte	Working Range (molL^−1^)	Response Time (min)	Remark	[Ref.]
CR-528	Spermine	3 × 10^−6^–1.2 × 10^−4^	30	A, ethanol solution	our work
	Spermidine	3 × 10^−6^–1.2 × 10^−4^			
	Ethanolamine	5 × 10^−5^–1 × 10^−3^			
	NaSH	2 × 10^−4^–3 × 10^−2^			
	2-ME	3 × 10^−3^–3 × 10^−1^			
CR-555	Spermine	2 × 10^−6^–2 × 10^−5^	30	A, ethanol solution	our work
	Spermidine	5 × 10^−6^–2.5 × 10^−5^			
	Ethanolamine	2 × 10^−5^–3.1 × 10^−4^			
	NaSH	1.2 × 10^−5^–2.5 × 10^−4^			
	2-ME	1.5 × 10^−4^–1 × 10^−2^			
4-methyl-7-azidocoumarin	NaSH	2 × 10^−5^, single concentration in a mechanism study	60	F, 50 mM PIPES, 100 mM KCl, pH 7.4	[24]
4-chloro-7-nitrobenzofura-zan (NBD-Cl)	H_2_S	~1 × 10^−6^–3.3 × 10^−5^	30	A, 50 mM PIPES, 100 mM KCl, pH 7.4	[25]
Fluorescent probe (HF-PBA) constructed from 3-HF and 2-(fluorine-2-yl-disulfanyl)benzoic acid (PBA)	H_2_S	~2 × 10^−6^–8 × 10^−6^	30	F, PBS (pH 7.4) containing 40% ethanol (*v*/*v*)	[26]
Coumarin–malonitrile conjugate	2-ME	2 × 10^−3^, single concentration in kinetics study	330	F, DMSO–HEPES buffer (1:2, *v*/*v*; 0.10 M pH 7.4)	[27]
Tyrosine-protected gold nanoparticles (Tyr-Au NPs)	Spermine, Spermidine	1 × 10^−10^–5 × 10^−5^	n.d.	A,F; PBS buffer at pH 6.0	[29]
Aggregates from poly[9,9-bis(6′-methyl imidazoliumbromide)hexyl)fluorene-co-4,7-(2,1,3-benzothiadiazole)](PFBT-MI) and surfactant	Spermine	0–1.2 × 10^−4^	n.d.	F, aqueous solution	[6]
Cu(II) complex of Schiff-base receptor organic nanoaggregates	Spermine	~2 × 10^−4^–1.4 × 10^−3^	3	A, DMF/water (1/99, *v*/*v*) solvent system	[43]

n.d.—not defined; A—absorbance-based assay; F—fluorescence-based assay.

## Supplementary Materials

The following are available online at http://www.mdpi.com/1424-8220/18/3/814/s1.
