# Supplementary material for: Design and Characterization of Dicyanovinyl Reactive Dyes for the Colorimetric Detection of Thiols and Biogenic Amines
Source: Sensors (Basel). 2018 Mar 8;18(3):814. doi: 10.3390/s18030814 (PMC5876516; doi:10.3390/s18030814)
Supplement: Supplementary file 1 [file sensors-18-00814-s001.docx]

Supplementary Material

Design and Characterization of Dicyanovinyl Reactive Dyes for the Colorimetric Detection of Thiols and Biogenic Amines

Tinkara Mastnak ^1^, Aleksandra Lobnik ^1,2^, Gerhard J. Mohr ^3^ and Matejka Turel ^2,^*

^1^ Faculty of Mechanical Engineering, University of Maribor, Smetanova 17, SI-2000 Maribor, Slovenia; tinkara.mastnak@um.si (T.M.); aleksandra.lobnik@um.si (A.L.)

^2^ Institute for Environmental Protection and Sensors, Beloruska 7, SI-2000 Maribor, Slovenia

^3^ JOANNEUM RESEARCH Forschungsgesellschaft mbH—Materials, Franz-Pichler-Straße 30, A-8160 Weiz, Austria; gerhard.mohr@joanneum.at

***** Correspondence: matejka.turel@ios.si; Tel.: +386-2-333-5663

|  |
| --- |
| (**A**) |
|  |
| (**B**) |

**Figure S1.** Spectral properties of CR-528 (**A**) and CR-555 (**B**) in different solvents;
([CR-528] = 2.4 × 10^−7^ M, [CR-555] = 2.6 × 10^−7^ M).

|  |  |
| --- | --- |
|  |  |

**Figures S2.** Spectral properties of CR-528 to biogenic amines (histamine, tyramine, cadaverine, putrescine); [CR-528] = 2.4 × 10^−7^ M, [biogenic amine] = 10^-2^ M.

|  |  |
| --- | --- |
|  |  |

**Figures S3.** Spectral properties of CR-555 to biogenic amines (histamine, tyramine, cadaverine, putrescine); [CR-555] = 2.6 × 10^−7^ M, [biogenic amine] = 10^-2^ M.
